# Local tumor control and toxicity in HIV-associated anal carcinoma treated with radiotherapy in the era of antiretroviral therapy

**DOI:** 10.1186/1748-717X-1-29

**Published:** 2006-08-18

**Authors:** Christoph Oehler-Jänne, Burkhardt Seifert, Urs M Lütolf, I Frank Ciernik

**Affiliations:** 1 Radiation Oncology, Zurich University Hospital, Switzerland; 2 Department for Social- and Preventive Medicine, Biostatistics, University of Zurich, Zurich, Switzerland

## Abstract

**Purpose:**

To investigate the outcome of HIV-seropositive patients under highly active antiretroviral treatment (HAART) with anal cancer treated with radiotherapy (RT) alone or in combination with standard chemotherapy (CT).

**Patients and methods:**

Clinical outcome of 81 HIV-seronegative patients (1988 – 2003) and 10 consecutive HIV-seropositive patients under HAART (1997 – 2003) that were treated with 3-D conformal RT of 59.4 Gy and standard 5-fluorouracil and mitomycin-C were retrospectively analysed. 10 TNM-stage and age matched HIV-seronegative patients (1992 – 2003) were compared with the 10 HIV-seropositive patients. Pattern of care, local disease control (LC), overall survival (OS), cancer-specific survival (CSS), and toxicity were assessed.

**Results:**

RT with or without CT resulted in complete response in 100 % of HIV-seropositive patients. LC was impaired compared to matched HIV-seronegative patients after a median follow-up of 44 months (*p *= 0.03). OS at 5 years was 70 % in HIV-seropositive patients receiving HAART and 69 % in the matched controls. Colostomy-free survival was 70 % (HIV+) and 100 % (matched HIV-) and 78 % (all HIV-). No HIV-seropositive patient received an interstitial brachytherapy boost compared to 42 % of all HIV-seronegative patients and adherence to chemotherapy seemed to be difficult in HIV-seropositive patients. Acute hematological toxicity reaching 50 % was high in HIV-seropositive patients receiving MMC compared with 0 % in matched HIV-seronegative patients (*p *= 0.05) or 12 % in all HIV-seronegative patients. The rate of long-term side effects was low in HIV-seropositive patients.

**Conclusion:**

Despite high response rates to organ preserving treatment with RT with or without CT, local tumor failure seems to be high in HIV-positive patients receiving HAART. HIV-seropositive patients are subject to treatment bias, being less likely treated with interstitial brachytherapy boost probably due to HIV-infection, and they are at risk to receive less chemotherapy.

## Background

The incidence of cancer of the anal canal is rising due to the increasing prevalence of HIV-infection and HPV-infection [1-4]. Standard therapy for invasive anal cancer is radiotherapy (RT) or chemo-radiation resulting in local tumor control (LC) rates and disease-free survival (DFS) in HIV-seronegative patients approaching 72 % and 73 %, respectively [5-7]. Few data exist on treatment outcome in HIV-seropositive individuals. Retrospective survival analyses of cohort patients in the pre-HAART era indicate that HIV-infection is associated with poorer outcome after combined chemo-radiation [7-10]. Though, some investigators reported lower doses of RT and chemotherapy being applied in patients with HIV-infection [3,11]. Side-effects tended to be more frequent and more intense in HIV-seropositive patients without HAART than in HIV-seronegative patients in some reports [12-14] whereas in others acute toxicity was moderate [15]. The increased likelihood of therapy-related toxicity correlated with low CD4 count in HIV-seropositive patients in the pre-HAART era in one report [16].

The introduction of HAART resulted in an increase of CD4 counts in responders and prolongation of survival. The influence of HAART on concomitant cancer treatment-related toxicity and treatment outcome of anal cancer remains controversial. Analysing very small patient cohorts, some authors showed no changes of the overall survival (OS) rates of anal cancer since the introduction of HAART [1] while others reported favorable treatment and toxicity outcome compared with results of the non-HIV population [17,18].

The aim of this study was to investigate clinical characteristics of HIV-seropositive and HIV-seronegative patients and whether the outcome in respect of treatment toxicity and survival after standard curative 3-D conformal RT with or without chemotherapy (CT) of invasive cancer of the anal canal is comparable between HIV-seropositive patients receiving HAART and stage and age matched HIV-seronegative patients.

## Patients and methods

### Patients

Ninety-one patients presenting with histologically proven invasive carcinoma of the anal canal between 1988 and 2003 at the Department of Radiation Oncology, Zurich, were treated with curative 3-D conformal RT alone or combined with CT. First, clinical characteristics, pattern of care and outcome of 81 HIV-seronegative patients were retrospectively analysed. Then, 10 consecutive HIV-seropositive patients receiving HAART (1997 and 2003) were retrospectively compared to 10 HIV-seronegative patients (1992 – 2003) matching for TNM-stage and age. Selection of matched HIV-negative patients was as follows: of 81 HIV-seronegative patients with invasive carcinoma of the anal canal, 42 patients matched for TNM-stage and of these 42 patients 10 patients corresponded for age and grading. After obtaining informed consent and internal institutional review approval, clinical outcome was analyzed by reviewing medical records and interviews of patients.

Pre-treatment staging was performed in all patients and included digital examination, endoluminal ultrasound, chest x-rays and either an abdominal ultrasound or CT scanning. Patients were staged according to the system adopted by the American Joint Committee on Cancer [19] and the Union International Contre le Cancer (UICC) before the primary treatment [19]. Post-treatment evaluation included a clinical examination including digital palpation at each visit and regular anal ultrasounds. Anoscopy and post-treatment biopsies were only performed when a suspicious lesion was identified.

### Treatment

No patient in the HIV-seropositive had primary radical surgery with colostomy compared with 5 HIV-seronegative patients (6 %). All patients except one HIV-negative patient that died during treatment completed curative 3-D conformal RT. Standard RT was administered over a five-week period to a dose of 45 Gy in 1.8 Gy per fraction followed either directly by an external beam radiotherapy (EBRT) boost or an interstitial high-dose rate (^192^Ir-HDR) boost after an interval of 2 – 3 weeks to deliver a total dose of 59.4 Gy. The fractionated HDR brachytherapy boost (14 Gy/7 fractions, thrice daily) was applied to patients with T1 – T3 tumors appropriate for interstitial treatment after EBRT of 45 Gy.

Chemotherapy consisted of fluorouracil (5-FU) and mitomycin-C (MMC). 5-FU was applied as a continuous infusion during the first five days of radiotherapy at a dose of 750 mg/m^2 ^or over four days at 1000 mg/m^2^. The cycle was repeated during week five of RT. MMC was given as an i.v. bolus on day one of radiotherapy (15 mg/m^2^) or twice during week 1 and 5 (10 mg/m^2^). When contraindicated, MMC was replaced by cisplatin given i.v., during 1 hour infusion, in week 1 and 5. Criteria for "non-adherence to chemotherapy" included omittance of chemotherapy or dose-reduction because of side effects.

### Toxicity

The common terminology criteria for adverse events v3.0 was used for assessing acute and late treatment toxicity [20]. Follow-up records addressing long-term toxicity were available for 95 % of the patients. Sphincter function was assessed by digital palpation.

### Statistics

Mean values are indicated with standard deviation. Differences between groups on continuous variables were tested using the Mann- Whitney test. SPSS version 12 was used with exact *p*-values. Fisher's exact test was used to test for differences between groups on categorical variables. Survival was calculated from the beginning of RT to the day of death or the date of last follow-up and time-to- recurrence from beginning of RT to the day of recurrence or date of last follow-up. Survival curves for the two groups were plotted according to the Kaplan-Meier method. Differences in survival across the groups were tested using the Log rank (Mantel-Cox) test.

## Results

### Results of HIV-seronegative patients

Mean age of the 81 HIV-seronegative patients was 61.6 +/- 12.7 years and 75 % of the patients were female (Table 1). Sixty-one patients (74 %) had a very good performance status (WHO 0°). Nine patients (11 %) had grad 1 and 3 patients (4 %) grade 2 WHO performance status and for 7 patients it was not known. At time of diagnosis, tumor stage distribution for T1/T2/T3/T4 was 15 %, 43 %, 31 % and 11 %. The majority of patients presented with nodal negative disease (61 %) and low grad tumors, i.e. G1 and G2 (62 %). Radical surgery with up-front colostomy was performed in 6 % of the patients. All of them received postoperative RT or chemo-radiation. RT-dose was 57.2 +/- 5 Gy and RT-duration was 53.6 +/- 17 days. Interstitial ^192^Ir-HDR brachytherapy boost was appropriate in 42 %. CT was applied to 72 % and included MMC in 64 % of all patients. After a median follow-up of 45 months OS at 10 years was 48 % and CSS was 75 %. Local failure rate at 10 years was 13 %. Eleven patients received salvage surgery resulting in a 10-year colostomy-free rate of 84 % (78 % if the 5 patients with primary radical surgery are included). Two of them had not achieved complete response to chemo-radiation. Severe acute side effects were relatively rare with 31 %. The most frequent severe side effects were dermatitis (17 %), diarrhea (6 %) or thrombopenia (12 %). Two patients died during or immediately after treatment due to cerebral bleeding under thrombopenia or cardiac failure. Chronic side effects could be evaluated in 54 patients (67 %). Thirteen patients (24 %) experienced either ulcera (2 %), chronic proctitis (11 %) or sphincter pressure impairment (15 %).

**Table 1:** Clinical characteristics, pattern of care and outcome of HIV-seronegative patients (n = 81). MMC = mitomycin-C, RT = radiotherapy, OS = overall survival, CSS = cancer-specific survival, m = months.

**Variable**	**HIV-seronegative patients**
**gender**	m:f = 20:61 (24.7%)
**age**	61.6 +/- 12.7 y
**WHO performance status I°**	14.8 %
**T1/T2/T3/T4**	14.8 %/43.2 %/30.9 %/11.1 %
**N0/N1/N2/N3**	60.5 %/17.3 %/11.1 %/8.6 %
**G 1/G 2/G 3**	8.6 %/53.1 %/27.2 %
**sphincter invasion**	25 (30.9 %)
**histology (basaloid)**	25 (30.9 %)
**radical surgery**	5 (6 %)
**chemotherapy**	58 (71.6 %)
**MMC**	52 (64.2 %)
**RT-dose**	57.2 +/- 5 Gy
**RT-duration**	53.6 +/- 17.3 d
**brachytherapy**	34 (41.9 %)
**follow-up (median/mean)**	45 m/51 +/- 34 m
**OS at 10 years**	48 %
**CSS at 10 years**	75.1 %
**acute side effects (3°)**	25 (30.9 %)
**skin**	14 (17.3 %)
**diarrhea**	5 (6.2 %)
**hematological **(% of MMC patients)	6 (11.5 %)
**infection**	3 (3.7 %)
**other**	1 (1.2 %)
**chronic side effects (n = 54)**	13 (24 %)
**ulcera**	1 (1.9 %)
**proctitis**	6 (11.1 %)
**sphincter pressure impairment**	8 (14.8 %)

### Results of HIV-seropositive patients receiving HAART and matched-pair analysis

#### Patient's characteristics

Mean age of HIV-seropositive patients was low with 44.5 +/- 10 years and most of them were male gender (90 %) (Table 3). All 10 HIV-seropositive patients were in good health. In patients with HIV-infection, median and mean CD4 counts were 266/μl and 303/μl (+/-241/μl), respectively (Table 2). HIV-seropositive patients presented with early stage (80 % T1/2), nodal negative (90 %) and well differentiated (80 % G1/2) tumors as shown in Table 3.

**Table 2:** Clinical characteristics of HIV-seropositive patients with access to HAART (n = 10). TNM: TNM classification of malignant tumors, HAART: Highly active anti-retroviral treatment. * indicates: HAART was started with or immediately before RT. CDC: HIV disease stage (Centers for Disease Control). RT: Radiotherapy. HDR: High-dose-rate brachytherapy. CT: Chemotherapy. 5-FU: Fluorouracil. MMC: Mitomycin-C. f/u: Follow-up. APR: Abdomino-perineal resection. NED: No evidence of disease. MD: Moist desquamation. TC-penia: Thrombocytopenia. 1 = stavudin, 2 = didanosin, 3 = lamivudin, 4 = zidovudin, 5 = efavirenz, 6 = abacavir, 7 = ritonavir, 8 = nelfinavir, 9 = amprenavir.

**TNM**	**CDC**	**CD4**	**HAART**	**RT (Gy)**	**RT duration (days)**	**CT (mg) 5-FU/MMC**	**acute toxicity**	**chronic side effects**	**recurrence**	**f/u**
T1N0M0	C3	80	1, 2, 7	54	45	16100		incontinence I°	local	APR, recurrence
T3N0M0	C3	847	1, 3	59.4	45	13600/34	MD		none	NED
T1N0M0	C3	64	2, 3, 6, 9	59.4	48	15400/15	MD, Tc-penia	skin III°	none	NED
T3N0M0	A3	88	3, 4, 5	59.4	47	14400/30		incontinence I°	none	NED
T2N0M0	A1	591	1, 2, 7	54	60	11250/40			loco-regional	palliation, failed
T2N0M0	B3	354	1, 2, 8 *	59.4	37	none	MD		none	NED
T2N2M0	C3	105	3, 4, 8 *	59.4	47	none			none	NED
T2N0M0	C3	262	1, 3, 8	59.4	56	6800/20	Tc-penia		inguinal	failed
T2N0M0	C3	190	3, 4, 6	55.8	43	none			local	APR, NED
T2N0M0	A2	370	3, 4, 5	55.5	46	15520/19	Tc-penia		none	NED

**Table 3:** Patient's characteristics of matched HIV-seropositive patients and HIV-seronegative patients matched for TNM-stage and age (n = 10 vs. 10).

**Variable**	**Study group**		**Fisher's exact test**
		
	**HAART-HIV +**	**HIV -**	
gender	m:f = 9:1 (90%)	m:f = 1:9 (10%)	0.001
age (mean)	44.5 +/- 10 y	45.2 +/- 7 y	NS
WHO-perform. Status I°	2 (20 %)	0	NS
T1	2	2	NS
T2	6	6	NS
T3	2	2	NS
N0	9	9	NS
N2	1	1	NS
G1	2		NS
G2	6	8	NS
G3		2	NS
Sphincter invasion	2 (22 %)	3 (30 %)	NS
Histology (basaloid)	2 (20 %)	4 (40 %)	NS
Mitomycin C	6 (60 %)	8 (80 %)	NS
Brachytherapy	0 (0 %)	6 (60 %)	0.005
RT-dose (mean)	57.7 +/- 2.4 Gy	58.5 +/- 3 Gy	NS

#### Pattern of treatment

No HIV-seropositive patient received radical surgery with up-front colostomy. Also in the matched HIV-seronegative cohort no patient underwent primary surgery. Total RT dose did not differ between HIV-seropositive and matched HIV-seronegative patients (57 – 58 Gy) whereas the duration of RT was longer in the matched HIV-seronegative cohort compared with the matched HIV-negative cohort (47.4 +/- 6 d vs. 59.8 +/- 10 d; *p *= 0.007). This was due to the interval between the EBRT and the brachytherapy boost used in HIV-seronegative patients. A significant difference was observed in the application of HDR-brachytherapy. No HIV-seropositive patient received HDR-brachytherapy boost compared with 60 % of the matched HIV-seronegative patients (*p *= 0.005). Adherence to chemotherapy seemed to be more difficult in HIV-seropositive patients with HAART than in the stage- and age-matched HIV-seronegative patients (30 % vs. 80 %, respectively; *p *= 0.08). Lack of adherence was either due to non-compliance, co-morbidity or concomitant medication, low CD4 counts, or severe thrombocytopenia before or during treatment.

#### Response and survival

**Figure 1:**
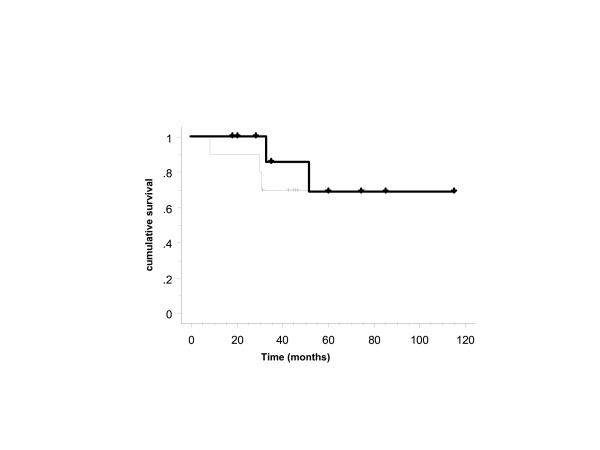
Overall survival of HIV-seropositive patients with access to HAART (n = 10) (thin line) and T-/N-stage, age-matched HIV-seronegative patients (n = 10) (thick line) with anal cancer (*p *= NS).

**Figure 2:**
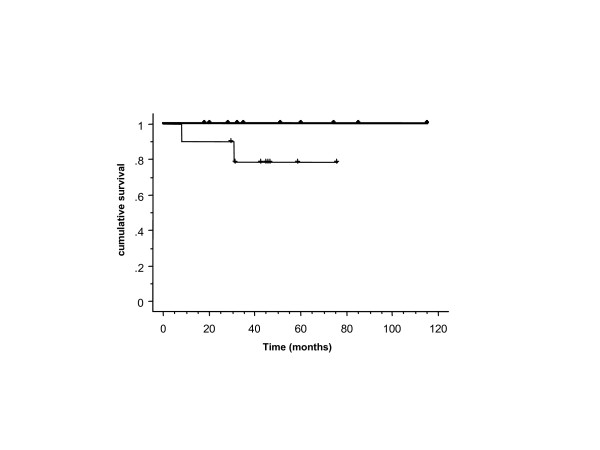
Cancer-specific survival of HIV-seropositive patients with access to HAART (n = 10) (thin line) and T-/N-stage, age-matched HIV-seronegative patients (n = 10) (thick line) with anal cancer (*p *= NS).

**Figure 3:**
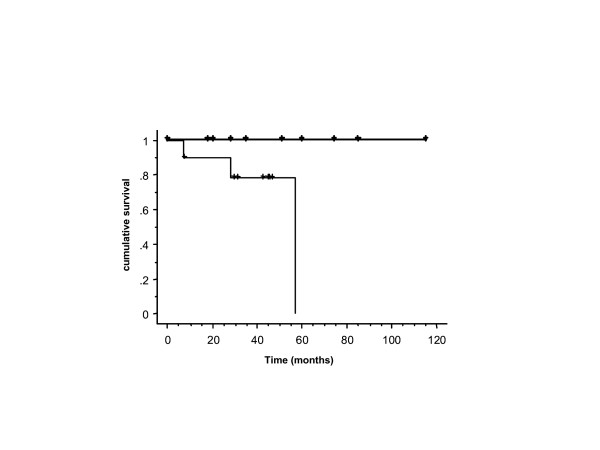
Time-to-local recurrence of HIV-seropositive patients with access to HAART (n = 10) (thin line) and T-/N-stage, age-matched HIV-seronegative patients (n = 10) (thick line) with anal cancer (*p *= 0.03).

Median follow-up was 44 months in the matched groups (HIV+: 8 – 76 months; HIV-: 19 – 116 months). Treatment with RT alone or combined with CT achieved complete remission in 100 % in HIV-seronegative as well as matched HIV-seropositive patients. Three HIV-seropositive patients with HAART suffered from local failure compared with no patient in the age-matched HIV-seronegative cohort, resulting in a statistically worse time-to-local recurrence (*p *= 0.03, Figure 3). Fifty-seven months after treatment, one HIV-seropositive patient suffered from a local relapse while no HIV-seronegative patient experienced local failure after 4 years. Colostomy-free survival was below 70 % (HIV+) and 100 % (matched HIV-). A non-significant trend towards impaired time-to-recurrence for HIV-seropositive patients was observed (4 recurrences in HIV-positive patients versus 1 recurrence in HIV-negative patients; *p *= 0.1). In HIV-seropositive patients, local recurrences were histologically confirmed in two cases. The two remaining patients suffered from locally extensive tumor disease (N = 1) or additional liver metastasis (N = 1). However, no significant difference in overall and cancer-specific survival was observed (Figure 1 and 2). The 1-year OS in the matched HIV-seropositive and -seronegative cohort was 90 % and 100 %, and the 5-year OS was 70 % and 69 %, respectively. The cause of death in young non-HIV individuals was predominantly secondary cancer. Two HIV-infected patients receiving HAART died of anal cancer compared to none of the HIV-negative control patients. In the present study, no HIV-seropositive patient with HAART died as a result of HIV-associated infections.

#### Acute toxicity

Although the frequency of acute grade 3/4 toxicities was doubled in HIV-seropositive individuals compared with matched HIV-seronegative patients (60 % vs. 30 %), this did not reach statistical significance because of low patient number. Therefore, a worse outcome in respect of acute toxicity can not be excluded. There was no grade 5 toxicity in the HIV-positive group or the matched HIV-negative group.

Cutaneous and gastrointestinal toxicity: Acute skin toxicity grade 3 was seen in 3 HIV-positive patients with HAART resulting in a rate of 30 % compared with 20 % of matched HIV-seronegative patients (*p *= NS). In the HIV-seropositive patients, no severe diarrhea was observed while it was reported in one HIV-seronegative patient.

Hematological toxicity: Three of six (50 %) HIV-seropositive patients receiving HAART who were treated with chemotherapy with MMC developed acute thrombocytopenia grade 3 (Table 1), compared to 0% of 8 matched HIV-negative patients. This resulted in a significant higher severe hematological toxicity rate (*p *= 0.05). Notably, two of the patients had CD4 count above 200 cells/μl.

#### Late toxicity

In respect of chronic side effects including chronic skin ulceration grade 3, complaints from chronic proctitis, sphincter muscle dysfunction and incontinence grade 1 no significant difference was found between HIV-seropositive and matched HIV-seronegative patients. Prolonged wound healing was seen in only 1 patient with HIV-infection (10%) without evidence of tumor persistence compared with no patient in the matched HIV-seronegative cohort. Though, this patient developed a secondary lymphoma at the site of ulceration which was treated with chemotherapy. One patient of each group developed sphincter pressure impairment after treatment. The rate of sphincter function preservation was around 70 % (HIV+) and 100 % (matched HIV-). One HIV-seropositive patient under HAART died 2.5 years after RT because of sigmoid colon perforation, an area outside the RT treatment fields.

## Discussion

HIV-infected individuals are confronted with a an increased risk for cancer disease, including anal carcinoma [1-4]. Although HAART has favorably influenced the incidence of non-Hodgkin lymphoma and Kaposis' sarcoma [21,22], the incidence of anal cancer has remained elevated in HIV-positive individuals [17,23-29]. HAART has a profound influence on a broad variety of organs and physiological systems resulting in improved function of the immune system, especially the CD4 T-cell compartment, and prolongs survival [30-40]. Protease inhibitors and non-nucleoside reverse transcriptase inhibitors are interfering with CT, because they are substrates and potent inhibitors or inducers of the cytochrome P450 (CYP) system [41]. Protease inhibitors may potentially act as radio- as well as chemosensitizers [42,43] putatively resulting in either better tumor control or increased toxicity.

This study is the first matched-pair analysis on anal cancer patients with HIV-infection receiving HAART comparing outcome with HIV-seronegative controls accounting for the three most important prognostic-factors T-stage, N-stage and age [44-46]. Several findings are of clinical importance. (i) Standard 3-D conformal RT alone or combined with standard CT can achieve complete tumor response rates of 100% in patients with HIV-infection under HAART. (ii) Despite the good response rates and maintained treatment with HAART, local failure rate is significantly higher when compared with the prognostically similar tumor-stage and age matched control cohort possibly compromising colostomy-free and cancer-specific survival. (iii) Nevertheless, disease-free and overall survival is similar in HIV-positive patients under HAART compared with HIV-negative patients. (iv) Standard chemo-radiation with mitomycin-C is associated with a high rate of severe acute hematological toxicity but not with increased long-term side effects in HIV-seropositive patients with HAART. (v) HDR-brachytherapy was applied less frequently to HIV-seropositive patients and adherence to chemotherapy was more difficult.

Since the introduction of HAART, one comparative [18] and six non-comparative retrospective reports have evaluated the outcome of HIV-seropositive patients with access to HAART [1,17,47-50]. Though, only 4 of these studies included more than 8 patients [1,17,18,50]. The study by Kinsella *et al*., presented at the Annual Meeting of the American Society for Therapeutic Radiology and Oncology (ASTRO) 2005, also compared consecutive HIV-seropositive patients receiving HAART with consecutive HIV-seronegative patients and concluded that standard combined chemo-radiation with 5-FU and MMC or cisplatin can achieve good tumor response (86 %) and DFS (79 %) in HIV-seropositive patients under antiretroviral therapy, similar to the HIV-negative cohort [18]. Tumor response was 100 % in our study and DFS at 5 years (70 %) compares similar to the results of Kinsella *et al*.. In contrast to the study by Kinsella *et al*., we observed an increased local recurrence rate in HIV-seropositive patients resulting in a colostomy rate of 30 %. Colostomy rate was not reported by Kinsella *et al*. The follow-up of our study was considerably longer with 44 months compared with 28 months in their study. It seems that a long follow-up time may be important in HIV-seropositive patients since in our study 1 HIV-seropositive patient had a local failure after 57 months which was not observed in the 81 HIV-seronegative patients. Importantly, HIV-seronegative patients in our study were TNM- as well as age- matched while in the study by Kinsella *et al*. the HIV-seronegative cohort included more advanced tumor stages (III and IV). Additionally, HIV-seronegative patients were an average of 17 years older than HIV-positive patients which may represent a different prognostic cohort. In respect of toxicity, they observed a low hematological severe toxicity rate in HIV-positive patients which is different to our study. This may be explained in part by the use of cisplatin instead of MMC. If so, cisplatin might be an advantageous alternative to MMC if applied at standard dose and not at high-dose like in the study by Blazy *et al*. [50]. Another study by Allen-Mersh *et al*. reported treatment and toxicity outcome after chemo-radiation in 46 HIV-seropositive patients with sufficient response to HAART [17]. After a median follow-up of 35 months, 1-year OS (85 %) was comparable to our results (90 %). In line with our study and not the study by Kinsella *et al*., they also noticed a high recurrence rate of 34 % after 1 year. In our analysis, local failure rate was 20 % (1 year) and 30 % (4 years). Due to toxicity, 22 % of the HIV-positive patients required RT breaks or altered chemotherapeutic regimens and 78 % of the patients experienced grade I-III toxicity. 70 % of patients were reported to receive MMC. In our study 60 % experienced acute grade III/IV toxicities and 50 % of the patients receiving MMC had severe thrombocytopenia requiring dose reduction. The importance of the CD4 count to toxicity of chemo-radiation was emphasized by Hoffman *et al*. who compared patients of the pre-HAART era in respect of CD4 count and found an increased rate of acute side effects associated with low CD4 counts [16]. The CD4 counts in the cohort of Allen-Mersh *et al*. may have been higher than the CD4 counts of the patients in the present trial. Notably, in the present study, two of the three patients with severe thrombocytopenia had a CD4 count above 200 cells/μl. Due to the small number of patients in the present study, no conclusions regarding CD4 counts are made. It is debatable, whether HAART contributed to the elevated acute toxicity. Some reports suggested a sensitizing effect of protease inhibitors on chemotherapy [42] or radiotherapy [43].

Results from randomized trials compare similar to ours regarding tumor response (100%), tumor progression at 1 year (20 %) and OS (70 % at 5 years). In the study by Bartelink *et al*. corresponding values were approximately 94%, 17 % and 56 % [51]. 4-year OS was 74 % in the trial by Flam *et al*. [6]. While colostomy-free survival reported was around 70 – 72 % in the whole patient cohort [6,51], in nodal negative and T1/2 tumors colostomy-free rate was 87 % and above 90 %, respectively [6]. Since our study included 80 % T1/2 and 90 % nodal negative tumors in HIV-seropositive patients, local tumor control rate and colostomy-free survival of below 70 % in HIV-seropositive individuals seem to be relatively poor and would be expected higher.

Thus, despite good tumor response and similar OS in our study, our data support the clinical experience that young HIV-seropositive patients with HAART and good performance status are prone to worse treatment outcome, a finding supported by Allen-Mersh *et al*. or the data of Blazy *et al*. who showed that high-dose RT (60 – 70 Gy) combined with 5-FU and cisplatin – given at a different regimen, though – resulted in grade 3 neutropenia in 4 of 9 HIV-seropositive patients receiving HAART (44%) [50]. Also, Bower *et al*. reported on 26 HIV-infected patients in the pre- and post-HAART era (16 patients with HAART) that the actuarial 2-year overall survival did not change after introduction of HAART [1].

There must be factors others than age or tumor stage for the higher local recurrence since the patients were well matched in respect of these prognostic factors. Such factors may be either patient-related or treatment-related. Patient-related factors may be persisting immunological alterations even after controlling HIV replication with HAART. Treatment-related factors may be the difficulties to apply full dose chemotherapy [52]. Chemotherapy and MMC in particular, was applied less frequently and with worse adherence in the HIV-seropositive patients either due to non-compliance, co-morbidity or concomitant medication, low CD4 counts, or severe thrombocytopenia before or during treatment. The importance of additional chemotherapy for the local tumor control has been demonstrated in several randomized trials [6,51,53-55]. Thus, we assume that chemotherapy is important for the local treatment success in HIV-positive patients, as well.

Another difference of treatment between seropositive and seronegative patients was the use of brachytherapy that was more reluctantly applied in HIV-seropositive patients. The stringent matching procedure for TNM-stage, age and grade to some extent did not allow an additional matching for brachytherapy. Though, retrospective analysis of 81 HIV-seronegative patients at our Department revealed no better outcome for patients receiving a HDR brachytherapy boost compared to patients with an EBRT boost. Due to missing randomized trials or retrospective comparative studies indicative for a definitive clinical benefit of brachytherapy, we lack evidence to encourage the use of interstitial treatment in HIV-seropositive patients with anal cancer [56-59]. In the present cohort, it is not clear whether brachytherapy has been used more reluctantly because of the HIV-infection harbouring a risk of complications and treatment-related death or because of other reasons, such as the particular anatomical presentation of the disease.

There are some limitations to the present study. Because of the low incidence only a small number of patients could be analysed with limited statistical power. Therefore, only clear and important differences were found. Due to the limited number of cases, this study must be considered hypothesis-generating and not conclusive. The case-control analysis improves the validity of the study results although there may still be a selection bias and despite that the controls were derived through a stringent selection algorithm, which did not allow changing matching cases before analysis. Furthermore, when the HIV-seropositive patients were compared with all, non-matched consecutive 81 HIV-seronegative patients, time-to-local recurrence was still inferior in HIV-seropositive patients under HAART. Matching for T-/N- stage as well as age is important since both are significant statistical parameters and because the demographics of HIV-patients with anal cancer differ from those of HIV-seronegative patients a fact not considered in the study by Kinsella *et al*.. Grading is not an independent risk factor when adjusted for stage [60] and gender can not generally be considered as a prognostic factor, although some multivariate analyses suggest that women may have a better prognosis than men [51,61-63]. The performance status was similar in all the patients investigated in this study, never below 80% of Karnofsky scale [46].

We conclude that HIV-seropositive patients with HAART and a good performance status are likely to achieve a complete response with standard 3-D conformal RT alone or combined with standard chemotherapy, although the risk for local relapse is high. Nevertheless, overall survival is comparable to HIV-seronegative patients. Despite good performance status the tolerability of chemo-radiation with mitomycin-C is limited by the toxicity of full dose chemotherapy. Therefore, in HIV-seropositive anal cancer patients with access to HAART, combined chemo-radiation is challenged to be more efficient at reduced toxicity at which cisplatin may represent a feasible option.

## Competing interests

The author(s) declare that they have no competing interests.
